# Reports of the Trustees, Steward, Treasurer, and Superintendent of the Insane Hospital, Augusta, Maine

**Published:** 1847-06

**Authors:** 


### PART III.—BIBLIOGRAPHICAL NOTICES.

ARTICLE XI.

*Reports of the Trustees, Steward and Treasurer, and Superintendent of the Insane Hospital.* 1845. Augusta, Maine.

This Institution, under the care of Dr. James Bates, appears from the reports before us to be in a prosperous condition.

There were in the institution at the opening of the year, seventy-six patients, and admitted during the year ninety-nine. Whole number one hundred and seventy-five.

Discharged during the year—

| | |
|---|---|
| Cured - - - - | 38 |
| Improved - - - - | 22 |
| Unimproved - - - | 23 |
| Died - - - - - | 7 |
| Making - - - - | 90 |

Leaving in the Hospital, at the close of the year, eighty-five patients.

We extract the following interesting paragraph:

"Although it is impossible to obtain correct information on a subject which many persons consider as one of delicacy, if not involving reputation of family, we have always sought it, in relation to hereditary predisposition. In some families this is very evident. In one case a father and two sons have been here; in two, the mother and daughter; in one, a man and his nephew and niece, at the same time. Of 486 admissions, 211 are represented by their friends as having insane ancestry or blood relatives in that condition.

"When we add to these, foreigners and others concerning whom little information can be obtained, there can be little doubt more than one half are hereditarily predisposed to that state of brain of which insanity is a symptom." E.

